# A Lipid-Bilayer-On-A-Cup Device for Pumpless Sample Exchange

**DOI:** 10.3390/mi11121123

**Published:** 2020-12-18

**Authors:** Yoshihisa Ito, Yusuke Izawa, Toshihisa Osaki, Koki Kamiya, Nobuo Misawa, Satoshi Fujii, Hisatoshi Mimura, Norihisa Miki, Shoji Takeuchi

**Affiliations:** 1 Artificial Cell Membrane Systems Group, Kanagawa Institute of Industrial Science and Technology, 3-2-1 Sakado, Takatsu-ku, Kawasaki, Kanagawa 213-0012, Japan; yoshi09tennis04@keio.jp (Y.I.); i.ysk626@gmail.com (Y.I.); tosaki@iis.u-tokyo.ac.jp (T.O.); kamiya@gunma-u.ac.jp (K.K.); nmisawa@azabu-u.ac.jp (N.M.); sfujii0331@gmail.com (S.F.); h-mimura@iis.u-tokyo.ac.jp (H.M.); miki@mech.keio.ac.jp (N.M.); 2 School of Integrated Design Engineering, Keio University, 3-14-1 Hiyoshi, Kohoku-ku, Yokohama, Kanagawa 223-8522, Japan; 3 Institute of Industrial Science, The University of Tokyo, 4-6-1 Komaba, Meguro-ku, Tokyo 153-8505, Japan; 4 Department of Mechanical Engineering, Faculty of Science and Technology, Keio University, 3-14-1 Hiyoshi, Kohoku-ku, Yokohama, Kanagawa 223-8522, Japan; 5 Department of Mechano-Informatics, Graduate School of Information Science and Technology, The University of Tokyo, 7-3-1 Hongo, Bunkyo-ku, Tokyo 113-8656, Japan

**Keywords:** lipid-bilayer membrane, sample exchange, nanopore sensor, portable sensor

## Abstract

Lipid-bilayer devices have been studied for on-site sensors in the fields of diagnosis, food and environmental monitoring, and safety/security inspection. In this paper, we propose a lipid-bilayer-on-a-cup device for serial sample measurements using a pumpless solution exchange procedure. The device consists of a millimeter-scale cylindrical cup with vertical slits which is designed to steadily hold an aqueous solution and exchange the sample by simply fusing and splitting the solution with an external solution. The slit design was experimentally determined by the capabilities of both the retention and exchange of the solution. Using the optimized slit, a planar lipid bilayer was reconstituted with a nanopore protein at a microaperture allocated to the bottom of the cup, and the device was connected to a portable amplifier. The solution exchangeability was demonstrated by observing the dilution process of a blocker molecule of the nanopore dissolved in the cup. The pumpless solution exchange by the proposed cup-like device presents potential as a lipid-bilayer system for portable sensing applications.

## 1. Introduction

Recently, biological membranes have been proposed for various sensing applications and have attracted considerable attention [1,2,3]. A representative example is a biological nanopore, which is a transmembrane protein that forms a nanometer-sized pore by reconstitution in a lipid-bilayer membrane. The principle of nanopore-based sensors is based on the interaction between the nanopore and analyte molecules, wherein the output signal is an ionic current through the nanopore that varies because of the presence of the analytes, such that the analyte is detected at the single-molecule level with a high sensitivity and specificity. The target analytes are mainly organic compounds and biomolecules—for example, biomarkers for diagnosis [4,5,6,7,8], toxic substances in food production, cosmetics, environmental monitoring [9,10], and illegal drugs and explosives for safety and security [11,12,13,14]. Because the core of the nanopore sensors is a single protein reconstituted in a lipid bilayer, the on-site sensing applications cited are expected using a miniaturized, portable device [15,16,17,18,19,20].

Solution exchangeability is an important characteristic for such a portable device to exchange the samples for series examination on the scene. Because a lipid bilayer formed in a device usually presents a poor physical stability [21,22,23], microfluidic channel components have been studied to gently exchange the solution facing the bilayer. However, the channel components including a pumping system may impede the simplicity of the sensor device, and the previous pumpless system took a relatively long time for solution exchange [24,25,26,27].

**Figure 1:**
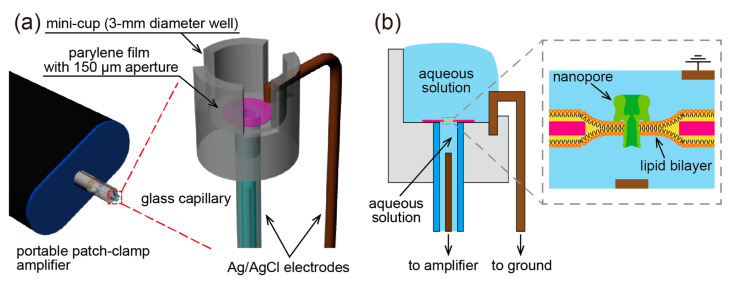
(**a**) Schematic of a lipid-bilayer-on-a-cup device named the mini-cup. The mini-cup device, mounted on a patch-clamp amplifier, consists of a 3 mm diameter well with a perforated polymer thin film at its bottom. The reverse side of the film is connected to the amplifier via a glass capillary. (**b**) Cross-sectional view of the mini-cup device. The well and the capillary are filled with aqueous solutions. At the aperture of the film, a lipid bilayer is formed and a nanopore protein is reconstituted. A pair of electrodes are used to monitor the ionic current through the nanopore.

**Figure 2:**
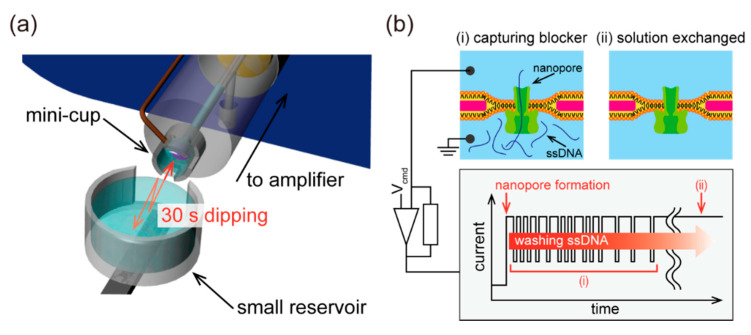
(**a**) Solution exchange procedure. A small reservoir, shown at the bottom, contacts the mini-cup device in a horizontal direction for 30 s, before moving apart. (**b**) Schematic diagram of a time course of an ionic current signal with solution exchange. Blocking events of a nanopore are observed with the existence of single-stranded DNA (ssDNA) (i), while the event frequency decreases with washing by solution exchange (ii).

In this study, we developed a device that integrates a lipid bilayer with a nanopore into a cup-like device, named mini-cup, which can steadily hold an aqueous solution to preserve the nanopore activity and rapidly exchange the analytes in the solution without additional equipment (e.g., a pump). Figure 1 illustrates the concept of the mini-cup device. The millimeter-sized cylindrical cup with slits is designed to hold an aqueous solution, at the bottom of which a lipid bilayer is formed with nanopore protein. The solution exchange can be conducted by simply fusing and splitting the solution in the cup with the solution of interest filled in a reservoir, as shown in Figure 2. Because of the small scale of the cup, the surface tension of the solution will be dominant over the gravity force. The use of an open well ensures that the solution in the cup is accessible to external solutions. Based on the aforementioned concept, we developed the mini-cup device. First, various slits were designed to simultaneously satisfy the capabilities of both the retention and exchange of the solution. The characteristics of the slit designs were examined using the mass or fluorescence intensity of the solution before and after the solution exchange. The exchangeability was further demonstrated by the translocation of single-stranded DNA (ssDNA) through a nanopore formed in the developed device.

## 2. Materials and Methods

### 2.1. Materials

A poly(methyl methacrylate) (PMMA) plate with a thickness of 4 mm was obtained from Mitsubishi Chemical Corporation (Kyoto, Japan). The dimeric precursor of the poly(chloro-*p*-xylylene) (parylene) film was purchased from Specialty Coating Systems Inc. (Indianapolis, IN, USA). Parylene film of 5 μm was obtained by chemical vapor deposition using a coater (PDS 2010, Specialty Coating Systems Inc., IN, USA). A glass capillary (G-1) was obtained from the NARISIGE Group (Tokyo, Japan). Silver wires for electrodes with diameters of 0.2 mm and 0.5 mm were purchased from The Nilaco Co. (Tokyo, Japan). Amphiphobic coating reagent (SFCOAT, SFE-B002H) was obtained from AGC SEIMI Chemical Co., Ltd. (Kanagawa, Japan). A UV-reactive glue (NOA 81) was purchased from Norland Products Inc. (Cranbury, NJ, USA). A phospholipid synthesized from 1,2-diphytanoyl-*sn*-glycero-3-phosphocholine (DPhPC) was obtained from Avanti Polar Lipids, Inc. (Alabaster, AL, USA). Nanopore-forming proteins (α-hemolysin) and *n*-decane were obtained from Sigma-Aldrich Co. (St. Louis, MO, USA). DNA oligonucleotides (polyT_50_) were purchased from Sigma Genosys (Tokyo, Japan). Other chemicals were obtained from Wako Pure Chemical Industries, Ltd. (Osaka, Japan) and Sigma-Aldrich Co. (St. Louis, MO, USA). All the reagents were used without further purification.

### 2.2. Mini-Cup Design

The mini-cup device consists of a well, a perforated parylene film, a glass capillary, and a pair of Ag/AgCl electrodes, as shown in Figure 1. A lipid bilayer was formed at a microaperture on the thin parylene film, and a nanopore was reconstituted in the bilayer. The parylene film divided two aqueous solutions, in which a pair of electrodes was placed, for monitoring the ionic current passing through the nanopore (Figure 1b).

**Figure 3:**
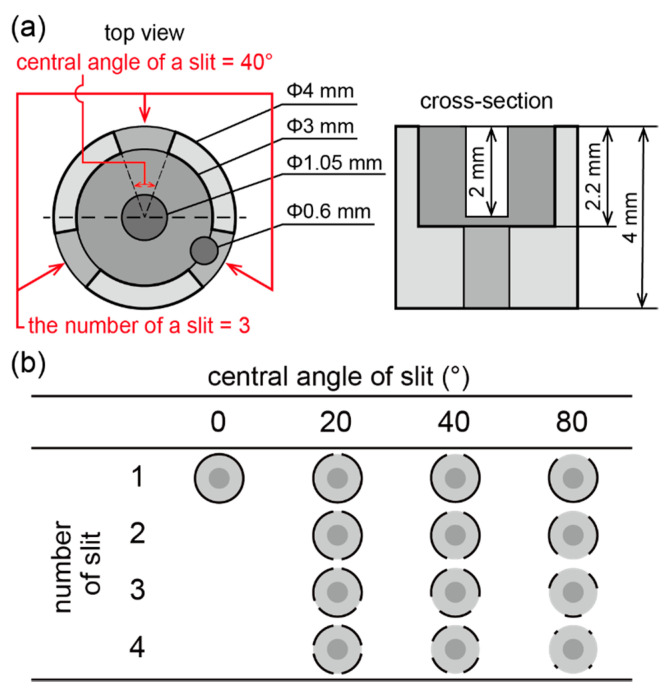
Mini-cup designs for optimization. (**a**) Description of the well dimensions. Note that the 0.6 mm diameter pocket was created to fix the ground electrode at the front side of the well (1 mm in depth). (**b**) Table of central angle and number of the slits of a mini-cup designed for the experiment.

The inner diameter and depth of the cup were 3.0 mm and 2.2 mm, respectively, to steadily hold the solution. Note that the Bond number of the water was below 1 at this scale (B_o_ = 0.3) [28]. Slits were additionally designed to enhance the solution exchangeability. For optimization, the slit design was varied and evaluated based on the capabilities of retention and exchange of a solution in the cup. Different central angles and number of slits were attempted for the mini-cup designs (Figure 3); for three angles of 20°, 40°, and 80°, four different mini-cups were fabricated with 1, 2, 3, and 4 slits, respectively (Figure 3b). A mini-cup without a slit was also used as a control. In Figure 3a, a 3-slit mini-cup with a central angle of 40° is shown as a representative. The well was 0.2 mm deeper than the slit to suppress the direct influence of the convection flow during solution exchange on the fragile bilayer membrane formed at the bottom of the well.

To evaluate the retention ability of respective mini-cup designs, the change in the volume of an aqueous solution was measured before and after the solution exchange. First, a mini-cup well was filled with 13 μL of a buffer solution (1 M KCl with 10 mM phosphate buffer, pH 7.0) and the mass was measured using an electronic balance (AP225W, Shimadzu Corporation, Kyoto, Japan). Next, the mini-cup well was contacted with the reservoir shown in Figure 2a, and the solution was fused with the external buffer solution (1 M KCl, pH 7.0) for 10 s. Then, the mini-cup well was gently separated from the reservoir, and the mass was measured again. The ratio of the mass of a solution was estimated from the measurements. The experiments were performed three times.

To estimate the exchangeability of a solution at respective mini-cup designs, the change in the sample concentration was observed before and after the solution exchange. First, a mini-cup well was filled with 13 μL of a buffer solution (300 nM calcein, 1 M KCl with 10 mM phosphate buffer, pH 7.0). Next, the solution in the mini-cup well was fused and rinsed with the external buffer solution (1 M KCl, pH 7.0) in the reservoir for 10 s. After the mini-cup well was gently separated from the reservoir, the exchanged solution in the mini-cup well was collected using a pipette and the fluorescence intensity of calcein was measured using a microscope (IX71, Olympus Corporation, Tokyo, Japan). The concentration was evaluated using the calibration curve shown in the Supplementary Materials (Figure S1). The experiments were performed three times.

### 2.3. Device Fabricatioin

**Figure 4:**
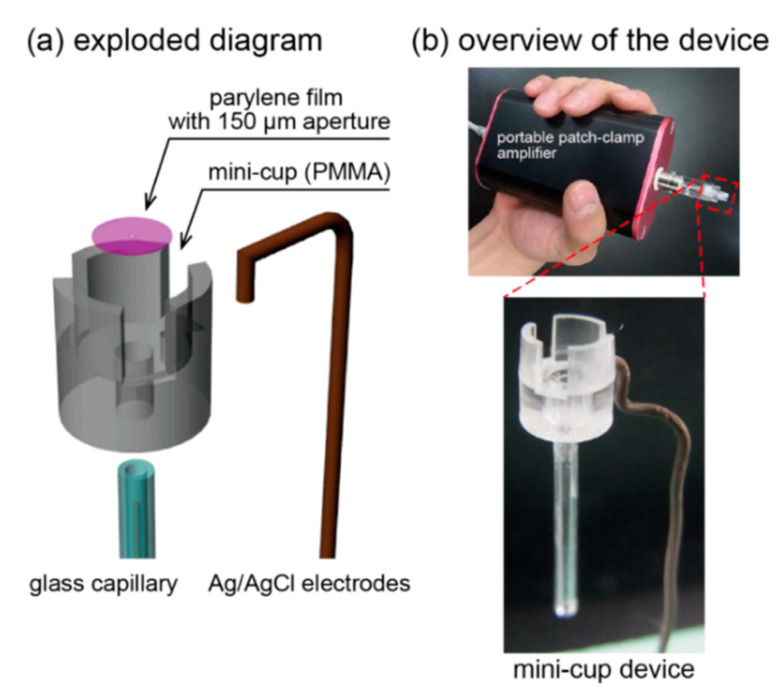
(**a**) Exploded diagram of the mini-cup device. (**b**) Photos of the developed device connected to a portable patch-clamp amplifier.

An exploded diagram of the device is shown in Figure 4a. A 150 μm aperture was formed on the thin parylene film (2 mm in diameter, 5 μm in thickness) using standard photolithography [29]. The well was fabricated from a PMMA plate by using an automated computer-aided design and computer-aided manufacturing modeling machine (MM-100; Modia Systems Co., Inc., Saitama, Japan). The inner surface of the well was then modified with an amphiphobic coating reagent, SFCOAT, to reduce the surface energy. Note that we previously confirmed that the stability of a solution in a small well was substantially enhanced by minimizing the size and decreasing the surface energy [28].

The mini-cup device was assembled as follows (Figure 4b): first, the perforated parylene film was fixed to the bottom of the well using UV-reactive glue (NOA 81). Then, a glass capillary was inserted and glued to the reverse side of the well. The Ag/AgCl ground electrode was affixed to the front side of the well. The Ag/AgCl working electrode was connected between the reverse side of the parylene film and a portable patch-clamp amplifier.

### 2.4. Lipid-Bilayer Formation and Nanopore Reconstitution

**Figure 5:**
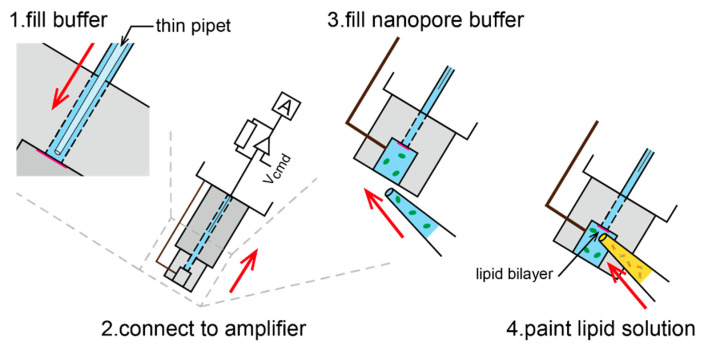
Procedure of a lipid-bilayer membrane formation on the microaperture in the mini-cup device. A painting method was used with 1,2-diphytanoyl-*sn*-glycero-3-phosphocholine (DPhPC) in *n*-decane (10 mg/mL) and a buffer solution (1 M KCl, pH 7.0).

We applied a painting method for lipid-bilayer formation at the microaperture of the parylene film [30]. As shown in Figure 5, first, the glass capillary was filled with a buffer solution (1 M KCl with 10 mM phosphate buffer, pH 7.0). Note that a thin pipette was inserted close to the parylene film to avoid air bubbles. The mini-cup device was then attached to the amplifier via an Ag/AgCl electrode inserted into the glass capillary. Second, a 13 μL aliquot of the buffer solution containing a nanopore-forming protein (α-hemolysin; 10 nM) was applied to the mini-cup well. Third, lipid-dispersed oil (10 mg/mL DPhPC/*n*-decane, approximately 0.5 μL) was infused close to the aperture. The lipid solution was gently swept across the aperture using a plastic needle, resulting in lipid-bilayer formation. Following bilayer formation, a nanopore was spontaneously reconstituted in the bilayer membrane (Figure 1b).

### 2.5. Ionic Current Recording with Solution Exchange

The ionic current between the Ag/AgCl electrodes was monitored using an in-house developed portable amplifier while applying direct current voltage [31]. The sampling frequency of the current trace was set to 5 kHz, and the obtained data were filtered by a Bessel low-pass filter at 1 kHz. We confirmed the nanopore reconstitution by observing the current trace. Note that the single nanopore presents a steady current with approximately 1 nS in 1 M KCl at neutral pH [32]. The current through the nanopore was partially blocked by the presence of ssDNA (polyT_50_) that obstructs or translocates the nanopore (Figure 2b) [33,34]. A more than 70% reduction in the nanopore current was defined as the blocking event.

Time-course monitoring of the ionic current signals through nanopore proteins was conducted using the mini-cup device to further demonstrate the solution exchangeability. A lipid bilayer was first formed at the aforedescribed microaperture. A 13 μL aliquot of the buffer solution containing a nanopore-forming protein (α-hemolysin; 10 nM) and ssDNA (approximately 10 μM) was added to the mini-cup well. The solution in the mini-cup was then exchanged with a buffer solution (1 M KCl with 10 mM phosphate buffer, pH 7.0) using a small reservoir (180 μL) that was horizontally in contact with the mini-cup well for approximately 30 s and separated from the mini-cup (Figure 2a). The current signals of the nanopore exhibited frequent blockades caused by ssDNA in the mini-cup well, as aforementioned. Because these blockades, which appear as temporal/partial current blocking, are proportional to the concentration of ssDNA, the blocking frequency should decrease as the solution is exchanged (Figure 2b). The blocking frequencies were estimated before and after the solution exchanges. All the data were analyzed using Clampfit software (Molecular Devices, SAN Jose, CA, USA).

## 3. Results and Discussion

### 3.1. Optimization of Mini-Cup Design

To optimize the slit design, both the retention ability and exchangeability of a solution in the mini-cup device were investigated. The retention ability was measured by the change in the solution volume held in the mini-cup well over the solution exchange; the volume should be constant before and after the exchange to specify the sample volume. The exchangeability of a solution is determined by the change in the analyte concentration over the solution exchange. Ideally, the concentration in the mini-cup well should be equal to the concentration in the external solution of interest by the exchange procedure.

**Figure 6:**
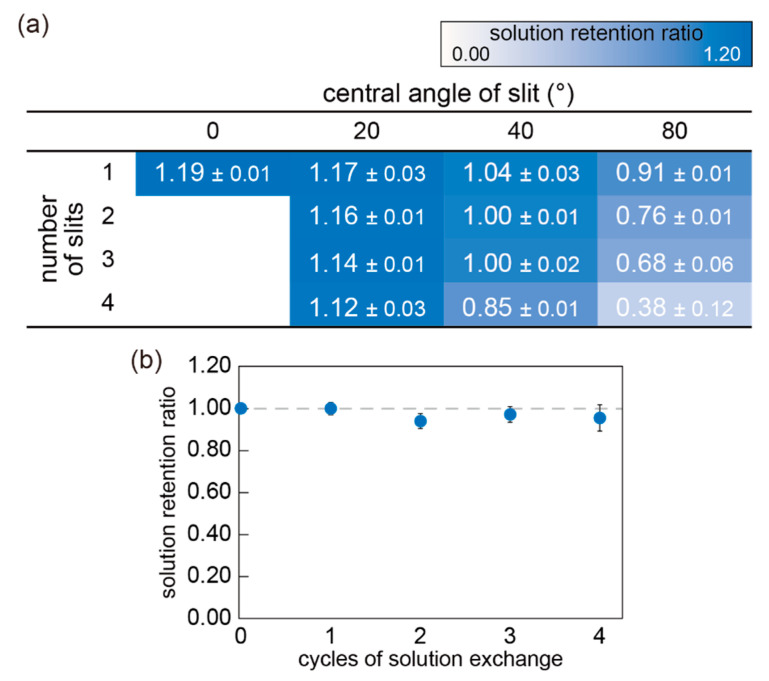
(**a**) Mass ratio of the solution held in mini-cup wells with different central angle and number of slits. The ratios were estimated from the mass change of the solution before and after the solution exchange (N = 3, mean ± standard deviation (SD)), respectively. (**b**) Retention ability over repetitive solution exchanges with the mini-cup well with 3 slits and 40° angle (N = 3, error bar: SD). For both (**a**,**b**), the solution exchange was performed by fusing the solution to the external solution for 10 s and in sequence separating them.

First, the retention ability was evaluated from the mass change of the mini-cup well before and after the solution exchange. As described in Section 2.2, 12 slit designs and a control of no slit were compared. Figure 6a shows the mass ratios of the solution in the mini-cup well with different slit designs. Values larger than 1 indicate that the solution volume in the mini-cup well increased by the solution exchange procedure, and vice versa. The results indicated that the retention ability decreased with an increase in the central angle and the number of slits. In the four slits with an angle of 80°, less than 40% of the solution remained after the single solution exchange. Without the slit, the solution increased approximately 20% from the well volume (13 μL) by the exchange. These results can be explained by the capillary rise between pillars or plates, in which the liquid height is inversely proportional to the spacing of the pillars or plates [35]. In the case of the mini-cup well, the capillary force became weaker with the increasing ratio of slit to perimeter, and the mini-cup lost the solution well by the exchange.

According to the results, the mini-cup wells of two or three slits with a 40° angle were the most appropriate designs regarding the retention ability because the ratio was close to 1. Figure 6b represents the repetitive exchange of a solution at the mini-cup of three slits with a 40° central angle. As the ratio remained close to one for four cycles of solution exchange, the well design guaranteed reproducible solution exchange with a constant sample volume.

**Figure 7:**
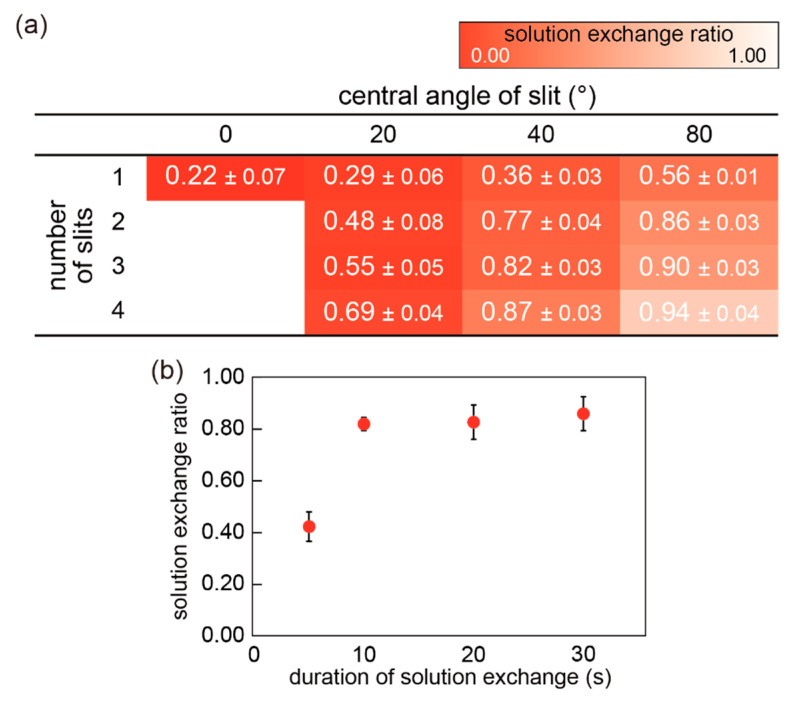
(**a**) Exchange ratio of the analyte concentration in mini-cup wells with different central angles and numbers of slits. The ratios and red colors in the table indicate the calcein concentration that remained in the well after the exchange (N = 3, mean ± SD). Concentration of calcein after the exchange was calculated from the fluorescence intensity using a calibration curve. (**b**) Exchange ratio vs. duration of the exchange procedure with the mini-cup well with 3 slits and a 40° angle (N = 3, error bar: SD). Each plot represents the single rinsing procedure with a defined duration.

Next, the exchangeability of a solution in the mini-cup was examined using a fluorescent molecule, calcein, dissolved in the solution; a decrease in the fluorescence intensity was observed after the 10 s rinsing procedure by exchange with a buffer solution (see Section 2.2). The exchange ratio was calculated as (C_0_-C)/C_0_, where C_0_ and C are the concentrations of calcein before and after rinsing, respectively. The concentration C was evaluated from the calibration curve obtained from the fluorescence intensity (see Supplementary Materials Figure S1). Figure 7 shows the exchange ratio of the solution in the mini-cup well with different slit designs. Values close to 1 indicate that the fluorescent molecule was thoroughly rinsed by a single solution exchange. The results indicate that the larger the slit ratio, the better the exchangeability. Without the slits, approximately 80% of the calcein remained after rinsing. With the slits, the solution exchange heavily relied on the mixing of the solutions between the mini-cup well and the reservoir, rather than diffusion accompanied by a concentration gradient. Note that the diffusion takes more than 100 min according to a one-dimensional model, as shown in Supplementary Materials Figure S2. We consider that the driving force behind the mixing is mainly attributed to forced convection arising from the impact of the fusion of the two solutions. This hypothesis is supported by the results in Figure 7a. The presence of the slits produces inlets and outlets of a convection flow triggered by the fusion of the solutions between the mini-cup and reservoir. Figure 7b shows the exchange ratios depending on the exchange duration, with the mini-cup well of three slits with a 40° central angle. The results indicate that the exchange ratio reaches a plateau in 10 s. It should be noted that the exchange time is faster than that of the previous pumpless system [25]. We consider that the mixing attributed to the convection flow ceases by 10 s after the fusion of solutions.

According to the before-described results regarding the retention ability and exchangeability for the slit designs, these two properties contradict each other. To determine the optimal slit design, we explored the minimum condition of the formula |1 − R| + |1 − E|, where R is the solution retention ratio and E is the solution exchange ratio obtained in Figure 6 and Figure 7. The slit condition of three slits with a 40° central angle appeared to be the best among the designs examined. Note that we weighed the two properties equally. In the following experiments, we applied the mini-cup device under this condition (three slits with a 40° angle).

### 3.2. Nanopore Formation Using the Mini-Cup Device

**Figure 8:**
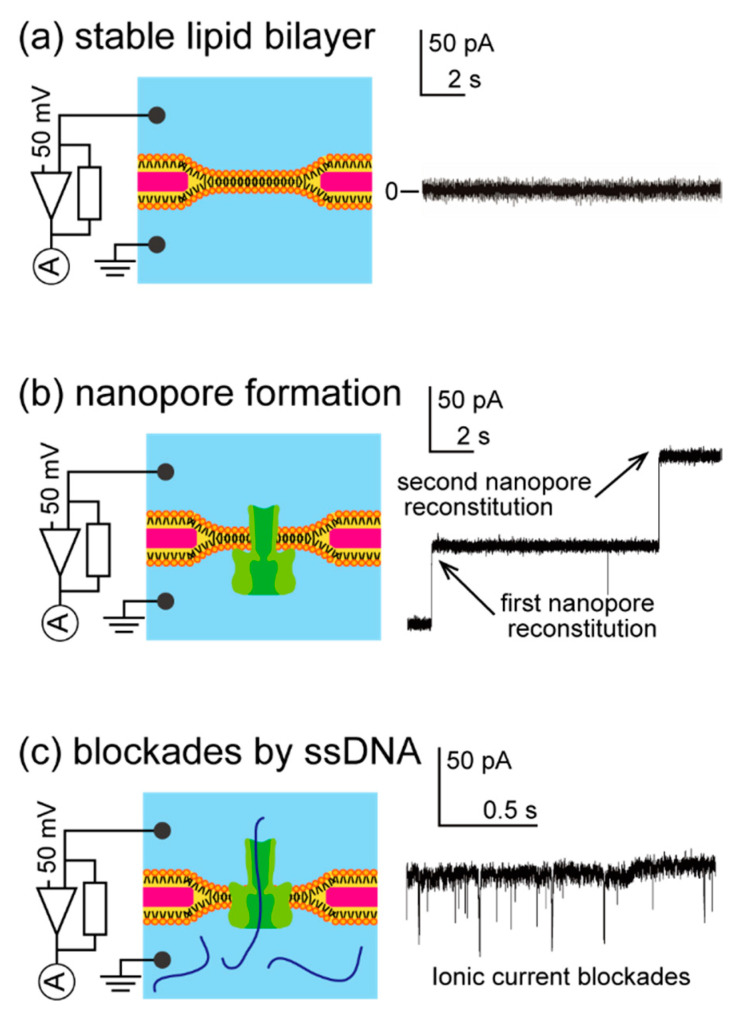
Ionic current traces with the mini-cup device. (**a**) Lipid-bilayer formation. (**b**) Reconstitution of nanopore proteins in a formed lipid bilayer. The reconstitution resulted in a stepwise increment of approximately 50 pA. (**c**) Blockades of ionic current by ssDNA. The interaction between the nanopore and ssDNA partially and temporarily blocked the open-pore current. Applied voltage: 50 mV.

We verified nanopore formation at a lipid bilayer in the mini-cup device using the slit design described in Section 3.1 and the ionic current traces shown in Figure 8. First, a lipid bilayer was formed by the painting method at the microaperture on the parylene film in the device using the procedure described in Figure 5. As a lipid bilayer generally prevents ion transport, a large resistance was observed with bilayer formation (Figure 8a). Next, the reconstitution of the nanopore protein α-hemolysin was monitored. As previously reported, α-hemolysin monomers dissolved in the buffer solution spontaneously reconstitute into a lipid-bilayer membrane and form a heptameric transmembrane pore with a 1.5 nm diameter at the constriction [36]. The conductance of the open pore is determined by the pore dimensions, ionic species in the solution, and pH. Figure 8b shows the time course of the ionic current accompanied by nanopore formation. The stepwise current was observed with a 1 nS conductance, which corresponds to nanopore formation at a lipid bilayer under the experimental conditions. These experiments were conducted using the mini-cup device that was held perpendicularly to the ground to further demonstrate the retention ability of the device by keeping the solution over the experiments. The results support the feasibility of the mini-cup design as a portable lipid-bilayer device.

A model sample of ssDNA (polyT_50_) was applied to the nanopore reconstituted in the mini-cup device to further confirm the relevance of the mini-cup as a portable bilayer device. The final concentration of 10 μM ssDNA was added to the mini-cup well. At the nanopore, ssDNA translocates or obstructs owing to the similarity of the pore size and the diameter of the ssDNA. These interactions of ssDNA with the nanopore hinder the ionic current passing through the pore, and produce spike-like current reductions of various durations and depths [34]. As shown in Figure 8c, we confirmed that the application of ssDNA to the mini-cup device partially and temporarily caused blockades of the open-pore current, indicating the interaction of the analyte with the nanopore sensor within the device. This result further demonstrated the applicability of the mini-cup device as a portable lipid-bilayer system.

### 3.3. Demonstration of Solution Exchange with Mini-Cup Device

We performed time-course monitoring of the ionic current and a solution exchange procedure to demonstrate the exchangeability of a solution in the mini-cup device. A nanopore protein was reconstituted in a lipid bilayer and ssDNA was also added in the mini-cup well in the beginning, as shown in Figure 8c. By the exchange procedure with a reservoir, the ssDNA and nanopore protein were rinsed away from the well (Figure 2b).

**Figure 9:**
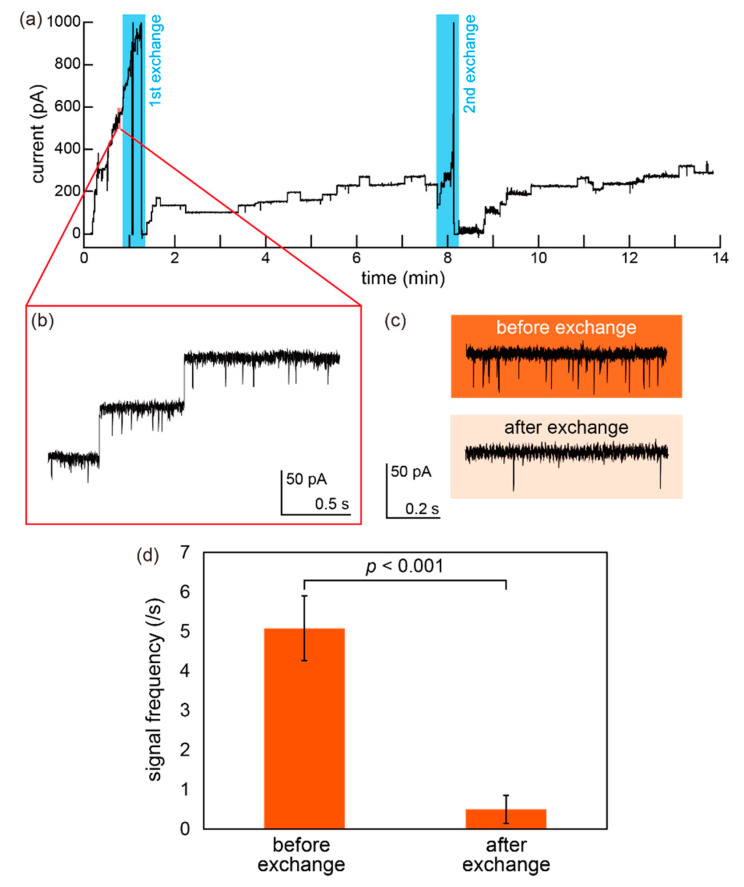
(**a**) Time-course monitoring of ionic current through the nanopore reconstituted in the mini-cup device. The solution exchange was conducted while maintaining the lipid bilayer. The light blue areas indicate the 1st and 2nd solution exchanges. Applied voltage: 100 mV. (**b**) Enlarged view of the current trace of serial nanopore formations in a lipid bilayer. (**c**) Typical blocking signals by ssDNA before and after the first exchange. (**d**) Frequencies of the blocking signals by ssDNA. In three individual experiments, the blocking signals were respectively collected for 30 s, and the frequencies were calculated. (N = 3, error bar: SD). The significance was assessed by *t*-test.

Figure 9 shows a representative current trace over the exchange procedures. More than 14 min of monitoring was possible without rupture of a lipid bilayer in this case. At the far left of the trace (close to time zero), a lipid bilayer was formed and nanopore proteins were serially reconstituted into the bilayer on the mini-cup device (Figure 9b). The initial concentration of 10 μM ssDNA in the mini-cup well was exchanged twice with a 1 M KCl buffer solution for 30 s using the reservoir. The exchanges are indicated with light blue areas. Note that the sharp peaks appearing during the exchange procedures in Figure 9a do not indicate the bilayer rupture but current noise due to external disturbance; however, because the survival rate of the bilayer was still low (18/32), the stabilization of the bilayer has to be improved [21,37]. Each stepwise increase in the current trace shows the reconstitution of single nanopores. The averaged time of the first nanopore insertion after a bilayer formation was 48 s (N = 3). We found that nanopore reconstitution rapidly and continuously occurs unless the α-hemolysin monomers are cleared from the solution in the well and the continuous nanopore formation generally causes a bilayer rupture within a few tens of seconds at a protein concentration of 10 nM [38]. The exchange of the solution, which removed the α-hemolysin monomers from the well, prevents unnecessary nanopore formation and preserves the device feasibility for sample detection with nanopores [24,25]. Solution exchange was also confirmed from the blockades by ssDNA. As shown in Figure 9c, a large number of blockades were observed before the rinsing (exchange procedure), whereas the number of blocking signals was significantly reduced by the first exchange. The frequency of the blocking signal is indicated in Figure 9d. After the second exchange, the blockades hardly occurred. These results indicate that the mini-cup device was able to maintain a nanopore in a lipid bilayer after the solution exchanges, demonstrating the possibility of a sample exchangeable device for the portable sensing applications of a lipid bilayer.

## 4. Conclusions

We developed a lipid bilayer-on-a-cup device for pumpless solution exchange. The device is composed of a millimeter-scale cup with vertical slits, and a lipid bilayer is reconstituted at the microaperture located at the bottom of the cup. The slit design was optimized to reproducibly hold a solution and efficiently exchange the solution with an external solution by a fusing-splitting procedure. Finally, we demonstrated the solution exchangeability of our device by replacing a blocker molecule of a nanopore with a blocker-free solution. Pumpless solution exchange with the mini-cup design would facilitate serial sample measurements using a nanopore sensor based on a portable lipid-bilayer system for on-site sensing applications.

## Supplementary Materials

The following are available online at https://www.mdpi.com/2072-666X/11/12/1123/s1: Figure S1: Correlation between fluorescence intensity and concentration of calcein; Figure S2: One-dimensional diffusion model.
