# Supplementary material for: A Lipid-Bilayer-On-A-Cup Device for Pumpless Sample Exchange
Source: Micromachines (Basel). 2020 Dec 18;11(12):1123. doi: 10.3390/mi11121123 (PMC7767076; doi:10.3390/mi11121123)
Supplement: Supplementary file 1 [file micromachines-11-01123-s001.pdf]

micromachines

MDPI

Supplementary Materials

A Lipid-Bilayer-On-A-Cup Device for Pumpless Sample Exchange

Yoshihisa Ito, Yusuke Izawa, Toshihisa Osaki, Koki Kamiya, Nobuo Misawa, Satoshi Fujii, Hisatoshi Mimura, Norihisa Miki and Shoji Takeuchi

S1. Correlation between fluorescence intensity and concentration of calcein

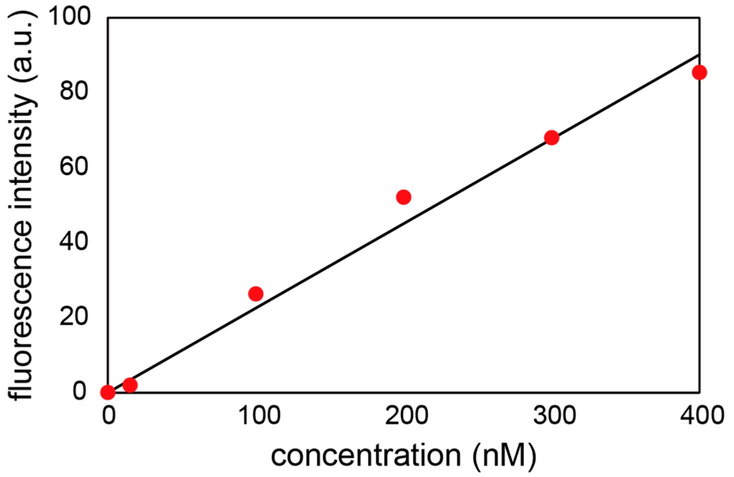

Figure S1. Correlation between fluorescence intensity and concentration of calcein. The fluorescence intensities of calcein at respective concentrations were measured using a fluorescence microscopy. The concentration inside of the mini-cup was then estimated using the calibration curve.

S2. One-dimensional diffusion model

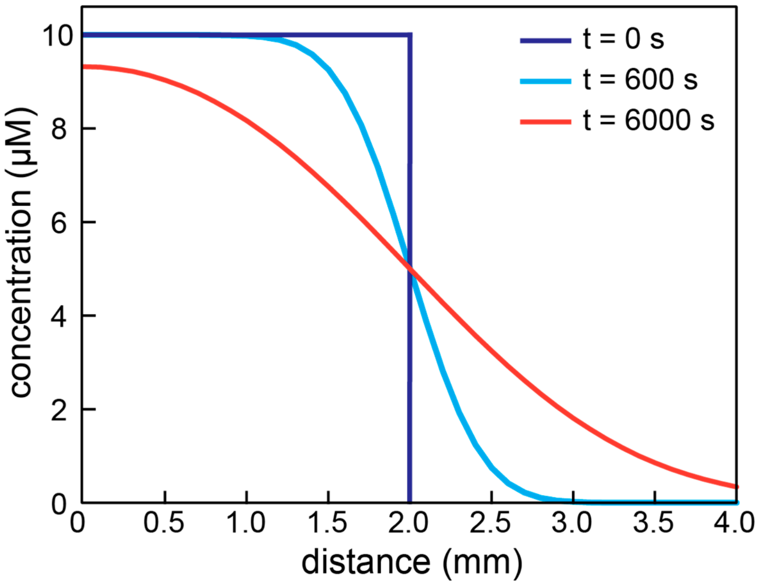

Figure S2. Diffusion of a molecule over time. A molecule held in a mini-cup ($0 < x < 1$, $C_0 = 10$ μM) diffuses by the fusion with a reservoir ($1 < x$). The model does not consider convection flow.

Crank showed one-dimensional diffusion model similar to our system.

$$C(x,t) = \frac{C_0}{2}\left[\mathrm{erf}\left(\frac{x+h}{2\sqrt{Dt}}\right) - \mathrm{erf}\left(\frac{x-h}{2\sqrt{Dt}}\right)\right] \quad (1)$$

where we applied the following parameters [1].

$$C_0 = 10\ \mu\mathrm{M} \quad (2)$$

$$D = 1.0\times10^{-10}\ \mathrm{m^2/s} \quad (3)$$

$$h = 2\ \mathrm{mm.} \quad (4)$$

1. CRANK, J. *The Mathematics of Diffusion*. Oxford university press: Oxford, England, 1975.

© 2020 by the authors. Submitted for possible open access publication under the terms and conditions of the Creative Commons Attribution (CC BY) license (http://creativecommons.org/licenses/by/4.0/).
